# An *In Vitro* Model of Latency and Reactivation of Varicella Zoster Virus in Human Stem Cell-Derived Neurons

**DOI:** 10.1371/journal.ppat.1004885

**Published:** 2015-06-04

**Authors:** Amos Markus, Ilana Lebenthal-Loinger, In Hong Yang, Paul R. Kinchington, Ronald S. Goldstein

**Affiliations:** 1 The Mina and Everard Goodman Faculty of Life Sciences, Bar-Ilan University, Ramat-Gan, Israel; 2 Department of Biomedical Engineering, Johns Hopkins University School of Medicine, Baltimore, Maryland, United States of America; 3 Singapore Institute for Neurotechnology (SINAPSE), National University of Singapore, Singapore; 4 Department of Ophthalmology, University of Pittsburgh, Pittsburgh, Pennsylvania, United States of America; 5 Department of Microbiology and Molecular Genetics, University of Pittsburgh, Pittsburgh, Pennsylvania, United States of America; University of Wisconsin-Madison, UNITED STATES

## Abstract

Varicella zoster virus (VZV) latency in sensory and autonomic neurons has remained enigmatic and difficult to study, and experimental reactivation has not yet been achieved. We have previously shown that human embryonic stem cell (hESC)-derived neurons are permissive to a productive and spreading VZV infection. We now demonstrate that hESC-derived neurons can also host a persistent non-productive infection lasting for weeks which can subsequently be reactivated by multiple experimental stimuli. Quiescent infections were established by exposing neurons to low titer cell-free VZV either by using acyclovir or by infection of axons in compartmented microfluidic chambers without acyclovir. VZV DNA and low levels of viral transcription were detectable by qPCR for up to seven weeks. Quiescently-infected human neuronal cultures were induced to undergo renewed viral gene and protein expression by growth factor removal or by inhibition of PI3-Kinase activity. Strikingly, incubation of cultures induced to reactivate at a lower temperature (34°C) resulted in enhanced VZV reactivation, resulting in spreading, productive infections. Comparison of VZV genome transcription in quiescently-infected to productively-infected neurons using RNASeq revealed preferential transcription from specific genome regions, especially the duplicated regions. These experiments establish a powerful new system for modeling the VZV latent state, and reveal a potential role for temperature in VZV reactivation and disease.

## Author Summary

Most adults worldwide harbor latent VZV in their ganglia, and reactivation from it causes herpes zoster. This painful disease is frequently complicated by long-term pain, neurological sequelae, or vision loss that require improved prevention and treatment strategies. Study of VZV latency and reactivation has been severely hampered by the inability to reproduce a persistent state *in vitro* or *in vivo* that can be experimentally reactivated. Our study establishes a system using human neurons derived from embryonic stem cells where multiple stimuli can induce reactivation from long term experimental latency. A potential role for temperature in VZV reactivation has been revealed with this system, which can now be used to study the latent/lytic switch of VZV for the first time.

## Introduction

Herpes Zoster, which results from reactivation of latent varicella zoster virus (VZV) is a common and debilitating disease that is frequently complicated by acute pain, diverse neurological sequelae, vision problems and difficult-to-treat chronic pain known as post-herpetic neuralgia. The VZV latent state is established in human sensory neurons of ganglia along the entire neuraxis during primary infection and disease, chickenpox. We know little of this state and how VZV reactivates from it to cause herpes zoster. Studies exploring VZV transcription in human dorsal root ganglia (DRG) removed post-mortem by methods such as *in situ* hybridization, northern blotting and RT PCR quantification, have suggested a limited VZV transcriptome (reviewed in [1],[2] and detection of VZV protein expression (i.e. [3],[4]) in latently-infected ganglia. However, the recent recognition that latent VZV genomes undergo viral transcription in ganglia following post mortem removal raised doubt as to what transcriptional events occur in the latent state [2]. Furthermore, reports of immunohistochemical detection of VZV proteins in sections from latently-infected ganglia has been confounded by non-specific staining, lipofuschin granules and antibody cross-reactivity with blood group antigens [5]. While no transcripts analogous to the non-protein coding latency associated transcripts (LATs) of the closely related herpes simplex viruses (HSV) have been found, VZV transcripts from other genomic regions has been reported [6],[7]. The most commonly reported transcript in human ganglia is that for ORF63 [7] (which has also been observed in rodent neurons in a model for VZV latency, i.e. [8], that encodes a transcriptional regulatory protein during lytic infection that may influence apoptosis and host cell survival [9],[10].

The events underlying the VZV latent state and reactivation from it have been difficult to decipher because of the lack of model systems of VZV latency and reactivation. In contrast to HSV, for which there are both small animal and *in vitro* models for latent infection that can be reactivated, there is no widely-used *in vivo* small animal model of latency or any *in vitro* system of persistent infection in which reactivation can be experimentally induced. Indeed, VZV has proven to be difficult to induce to reactivate, even from post-mortem human ganglia harboring latent VZV genomes. The strict human specificity of VZV has precluded the use of most rodents as models of latency because no animal model reproduces human disease and most rodents do not even support VZV replication. A possible exception is the guinea pig, and VZV infection of enteric neurons *in vitro* [11] and a new *in vivo* model of enteric neuron infection [12] have been proposed as potential models for VZV latency. However, it is possible that data obtained from it may not extend to human ganglionic latency due to species differences.

Human dorsal root ganglia tissue transplanted to SCID mice have been pioneered for study of VZV neuronal infection by Arvin and colleagues (reviewed in [13], [14]). Human DRG obtained from 2^nd^ trimester fetuses can be infected with VZV administered either directly into the fetal DRG graft, or following venous administration of VZV infected human T-cells. VZV in the graft initiates a productive infection in neurons and satellite glial cells for several weeks, but then enters a state in which viral genomes are retained up to 56 days after infection without apparent productive replication. Low levels of transcripts from the ORF63 genomic region were detected in this model system, but reactivation of VZV in the model has not yet been documented. As a closed system of a complex tissue, it is not amenable to real time evaluation of VZV infection and reactivation. It is also hampered by the limited supply of fetal tissues, expense and being a technically demanding system. For these reasons, the modeling of latency *in vitro* with human neurons would be highly beneficial to the field.

Several *in vitro* models of using cells of human origin have therefore been proposed for studying for VZV neuronal replication and persistence. These include the use of SH-SY5Y neuroblastoma derived neuron-like cells [15], and human neurons obtained from fetal DRG or differentiated from stem cells (hESC, iPSC and neural stem cells, i.e. [16],[17],[18],[19]. Some have been shown to host a productive VZV infection, while others host an apparently non-productive infection in which the genome is maintained for prolonged periods [19],[20]. We recently presented evidence that the different outcomes of VZV infection is partly influenced by the quantity of virus used to infect the cultures [21], although lack of spread of infection observed in some models complicates the interpretation of what kind of infection the persistence actually reflects. One report in which persistent non-productive VZV infection of iPSC-derived neurons occurred, presented evidence for widespread transcription suggestive of an abortive type of infection, rather than a persistent quiescent state [6]. We argue that a compelling experimental model of neuronal VZV latency requires the ability of the cells to be able to fully support VZV infection and spread upon experimental reactivation. This has, to our knowledge, not yet been achieved.

We present here an extension of our previously described hESC-derived neuron model system, for which we have established conditions that lead to a prolonged, non-productive neuronal VZV infection that can be experimentally reactivated. Persistent VZV infections can be established either using acyclovir to block lytic infections, or by infection of axons without use of antiviral drugs. Persistently-infected human neuronal cultures maintain viral genomes for up to two months, with minimal transcription and undetectable translation of several VZV proteins. Importantly, renewed replication of VZV genomes and virus protein production can be initiated experimentally by interfering with growth factor-PI3 Kinase signaling cascades. We further show that stimulation combined with incubation at a reduced temperature (34°C) results in a productive, spreading infection. Comparison of the transcriptomes of quiescently vs productively-infection human neurons by RNAseq analysis reveals a preferential RNA transcription of select genomic regions during latency. In particular, the short repeated regions of the VZV genome encoding the regulatory proteins IE62 and IE63 are enriched for transcription in persistently-infected neurons. This model system should permit the elucidation of biology of VZV reactivation in detail that has not been possible until now.

## Results

### Human embryonic stem cell (hESC)-derived neurons support a non-productive, persistent VZV infection

**Fig 1:**
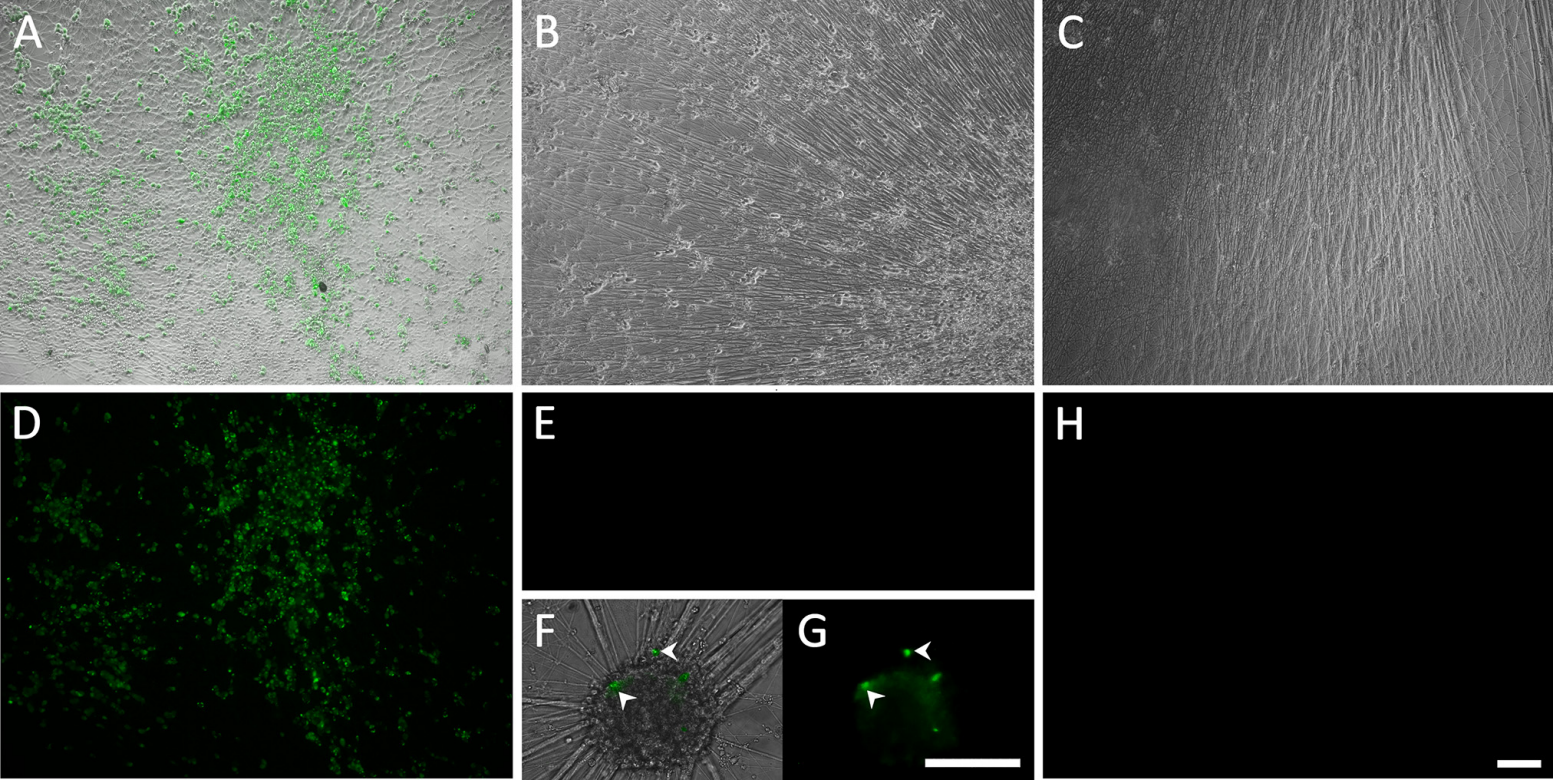
Living cultures of hESC-derived neurons infected with VZV-GFP66. (A&D) High PFU cell-free VZV-GFP66 infects most neurons in cultures productively as seen by GFP expression. B-H. Neuronal cultures treated with acyclovir (ACV) (see methods) and infected with low PFU VZV-GFP66 2 weeks (B, E-G) and 7 weeks (C&H) post infection. In about 50% of the wells no GFP+ cells were observed (E&H), in the other half of wells GFP+ neurons appeared as individual cells (F&G, arrowheads) and 1–5 small (<10 cell) clusters after 3–4 days. The diffuse green in G is autofluorescence of neurofuscin and not GFP A-C and F are merges of fluorescence and phase-contrast images showing GFP and the neurons, and D,E,G-H) show fluorescence-only micrographs for GFP expression. Scale Bar = 100μm.

We have previously reported that hESC-derived neurons exposed to high MOI cell-free recombinant pOKA-derived VZV with fluorescent protein reporters of viral protein expression, results in a spreading, productive infection [21]. In order to obtain non-productive persistent VZV infections in hESC-derived neurons, we exposed them to low PFU (0.001 MOI) of cell-free VZV in the presence of acyclovir (ACV) for 6 days. To detect productive infection, we used VZV66GFP [22] where GFP is fused to the N terminus of ORF66. GFP-tagged ORF66 has been shown to be functional, and as a presumed early gene, it should report productive (lytic) infection events, including those associated with reactivation. Using this approach (shown schematically in S1 Fig), in approximately half of neuron-containing wells exposed to VZV individual ORF66GFP+ neurons appeared in 1–5 small clusters, while the remainder did not contain any GFP+ cells (Fig 1). By comparison, infection of parallel cultures with higher levels of cell free VZV (MOI 0.02) resulted in GFP expression in many neurons by 3 dpi and an obvious productive, spreading infections. After 6 days we removed the ACV from the low MOI exposed cultures, and maintained them in its absence up to 7 weeks. All wells that were GFP-negative at the time of ACV withdrawal remained GFP-negative, strongly suggesting a lack of spontaneous reactivation.

We conjecture that the combination of ACV treatment and low MOI conditions are at the threshold for generating a productive infection, resulting in ½ the wells containing GFP+ cells that overrode the ACV inhibition, while the other half were effectively prevented from expressing ORF66. No visible cytopathic effect was observed in these GFP- wells throughout the experimental period. S1A Table indicates the numbers of GFP+ and GFP- negative wells we observed in our initial 7 experiments. Since our interest was only in studying events in the wells not containing GFP+ neurons, we eliminated GFP+ wells from further analysis.

### hESC-derived neurons exposed to VZV in the presence of ACV contain viral DNA and transcripts

**Fig 2:**
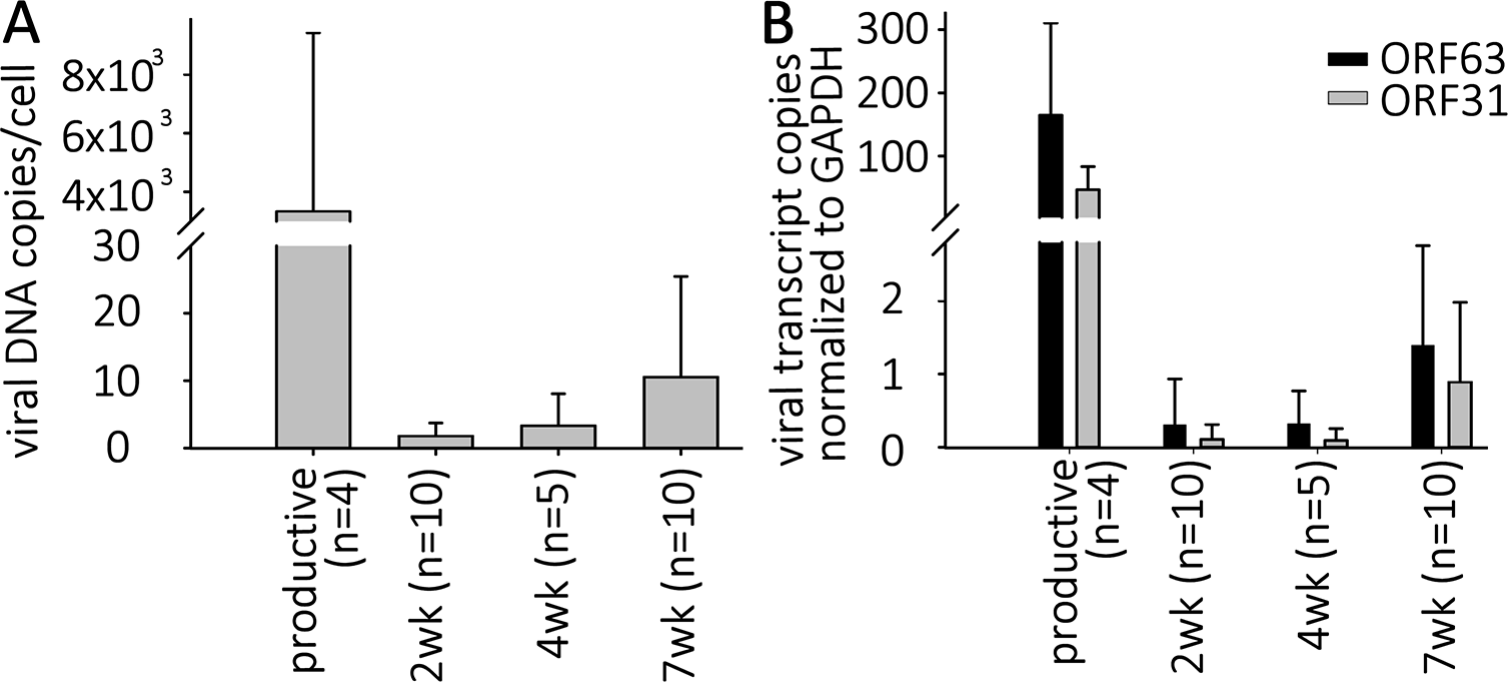
VZV DNA and transcripts are present in hESC-derived neurons productively or quiescently infected with VZV. Neurons were infected with high or low MOI VZV-ORF66-GFP in the presence of acyclovir, and ACV removed after 6 days incubation. (A) DNA and RNA were extracted from the GFP negative wells 2, 4 or 7 weeks after infection. Levels of VZV genomes and transcripts for ORF63 and ORF31 (gB) were quantified using Taqman probes and digital qPCR and normalized to GAPDH. (B) Transcripts levels detected from both ORFs in quiescently and productively-infected neurons, showing much higher levels in productively-infected neurons.

While the establishment of these GFP-negative cultures used a low level of virus, sufficient virus per well was added that would be expected to infect at least a fraction of neurons [18]. To address if VZV genomes were present in ACV treated neurons, wells devoid of GFP+ neurons were assayed for VZV DNA and transcripts using Taqman digital qPCR for ORF63 and ORF31 and compared to productively infected (GFP+) wells. Viral DNA was detected in all GFP-negative wells, with DNA copy numbers at 2 and 4 wk pi calculated to be approximately 2 and 3 copies per cell, respectively. By contrast, wells containing GFP+ neurons infected productively with high MOI cell-free VZV contained more than 1000x more copies of the VZV genome (Fig 2A). We conclude from these data that hESC-derived neurons exposed to VZV under these conditions maintain VZV genomes without productive infection, similar to the state of latency.

**Fig 3:**
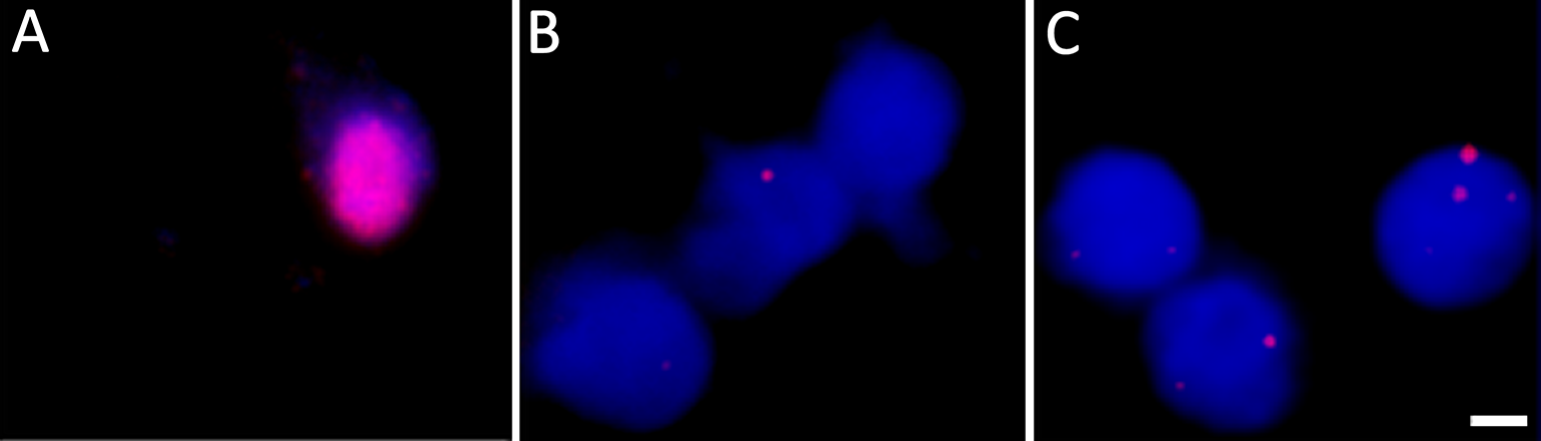
DNA fluorescent *in situ* hybridization confirms the presence of VZV genomes in nuclei of hESC-derived neurons quiescently infected with VZV. Neurons were infected with VZV-ORF66-GFP and *in situ* hybridization performed on isolated nuclei as described in the methods. (A) shows FISH of nuclei from productively and (B) from quiescently infected neurons. Almost all quiescently, infected FISH+ neurons contained only one puncta in their nuclei. (C) FISH for VZV genomes in quiescently infected neurons receiving the PI3K inhibitor LY as a reactivation stimulus. After LY treatment there was a slight decrease in the percentage of FISH+ nuclei in the preparations, but 25% of labeled nuclei contained 2 or more puncta, suggesting additional sites of VZV genomes had appeared. See Table 1 for quantification of the puncta. Scale Bar = 5 μm.

Simultaneous digital qRT-PCR analyses of viral transcript levels for ORF63 (IE63) and ORF31 (gB) in GFP-negative wells detected low levels (Fig 2B), whereas wells containing GFP+ neurons contained transcripts for ORF63 and ORF31 at levels greater than three orders of magnitude higher, consistent with the higher levels of genomes. These results suggest that transcription of not only IE, but also late genes continues from persistent VZV genomes without viral protein expression (as represented by the essential kinase ORF66 detected by GFP fluorescence) in these neurons (see RNASeq results below). This contrast to what has been reported in PCR studies of cadaver ganglia, where transcription appears to be limited to ORF63 [7],

While qPCR results suggested approximately 2–3 genomes per cell in the cultures, this technique does not permit the differentiation of a large number of neurons containing few VZV genomes from a few neurons containing higher copies of genomes. We therefore performed fluorescent *in situ* hybridization for VZV DNA (DNA-FISH) to estimate the number of neurons harboring quiescent VZV genomes. In nuclei of productively infected neurons, the hybridization signal for viral DNA fills most of the nucleus, consistent with viral replication and nuclear modifications induced by VZV infection (Fig 3A). In contrast, VZV hybridization signal at 2 wk pi (one week after removal of ACV) was present in approximately 4% of neuronal nuclei from GFP negative cultures, with the signal appearing as small fluorescent puncta (Fig 3B), reminiscent of puncta observed in neurons hosting latent HSV genomes [23]. Most labeled nuclei contained only a single hybridization punctum (Table 1). Given that DNA levels are at a few copies per cell in the population, each *in situ* hybridization punctum represents multiple VZV genomes in a single neuron.

Similar analyses were carried out on longer term cultures maintained without the appearance of GFP expression or cytopathic effects for 7 weeks. Viral DNA content in these cultures, measured both by qPCR and FISH, showed that there was a continued presence of low levels VZV genomes and a similar proportion of neuronal nuclei harboring them, although the levels were more variable (Fig 2A and Table 1).

**Table 1:** Percentage of neuronal nuclei containing fluorescent puncta following DNA FISH to detect VZV genomes.

Number of fluorescent foci	1	2	3	>3
2 wk post infection	97.5	2.5	0	0
2 wk post infection after LY treatment	74	6	8	12

Analysis of viral transcripts in quiescently infected neurons for the 10 experiments maintained for 7 weeks after ACV withdrawal revealed a great deal of variation between repetitions. These long-term experiments could be divided into two groups of 5. In one group, very few viral transcripts were detected by PCR for the two VZV genes (0.003 copies/cell of ORF63, 0.001 copies per cell of ORF31), while the in the second group of 5, there were transcripts at three orders of magnitude higher levels higher. Since all the longer-term neuronal cultures remained GFP-negative at the time of harvest, we hypothesize that the increase in VZV transcripts in one group of these extended cultures reflects partial release of repression of viral gene expression without progression to lytic replication and detectable GFP-ORF66 protein expression.

After careful and repeated attempts using several different antibodies, we did not detect ORF63, ORF62, gI or GFP (reflecting the ORF66 protein) protein expression by immunocytochemistry in quiescently infected hESC-derived neurons. However, our cultured human stem-cell-derived neurons, like neurons in peripheral ganglia of humans and rodents, contain lipofuschin granules that are autofluorescent, and the presence of this autofluorescence may prevent unequivocal detection of very low levels of VZV proteins.

### Reactivation of neurons quiescently infected by VZV by growth factor withdrawal

**Fig 4:**
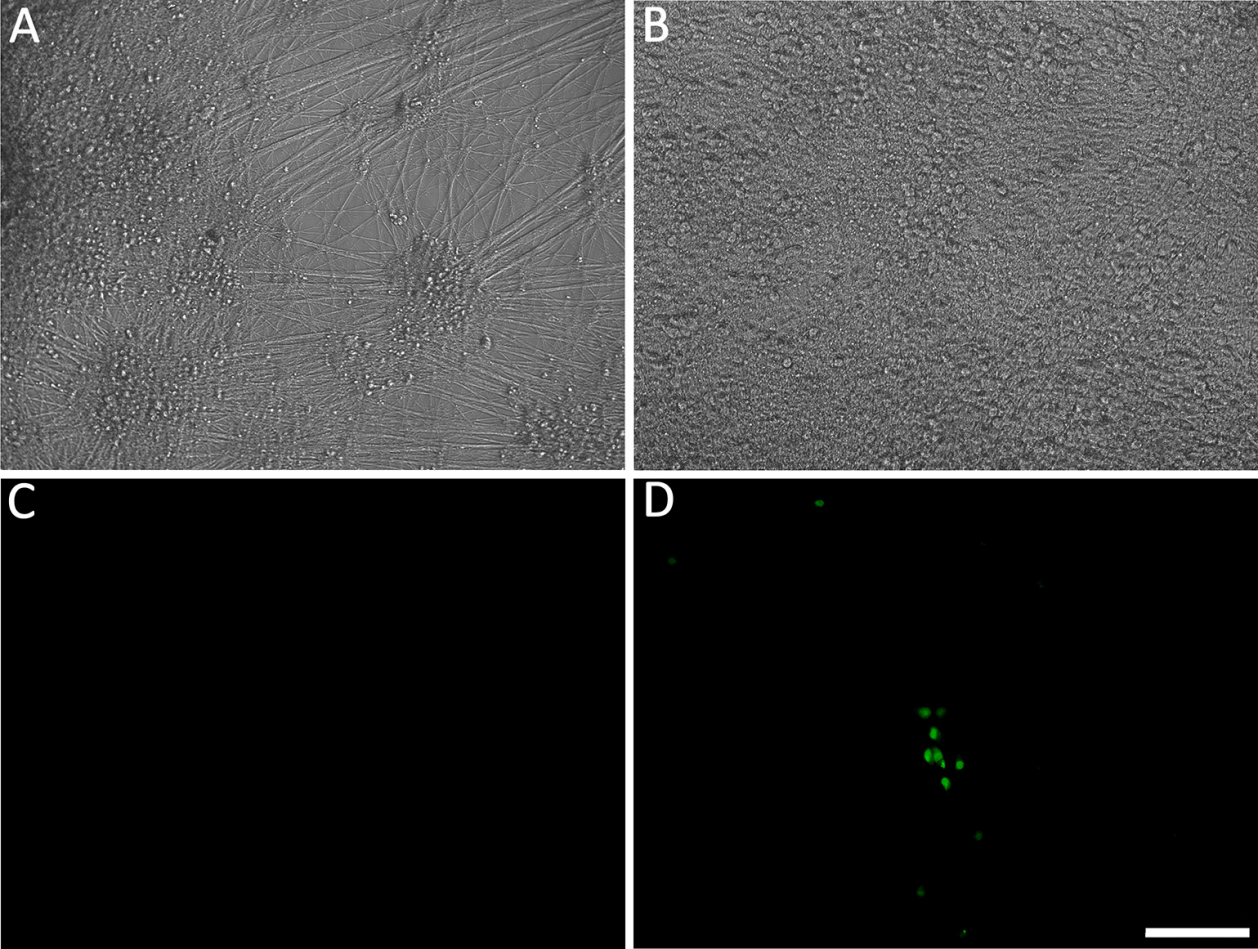
Reactivation of quiescent VZV in hESC-derived neurons induced by growth factor withdrawal. (A&C) hESC-derived neurons were incubated with low MOI VZV in the presence of ACV. Two weeks after exposure to virus and one week after removal of ACV, no GFP expression was detected in 50% of neuron-containing wells. At this time point, growth factors (GF) were withdrawn from the medium in wells that were GFP negative. By 4 days after GF withdrawal, massive loss of neurites was observed (B), eventually resulting in death of the cells in the wells by day 5 after treatment. However 30% of the initially GFP negative wells receiving GF-withdrawal treatment contained single and small foci of ORF66 protein-expressing neurons. (D). A and B are phase micrographs, C and D fluorescence micrographs of the same microscopic fields. Scale bar = 100μm.

It was argued above that a valid model system for the VZV latent state should be experimentally reactivatable. We therefore evaluated stimuli for their ability to drive the re-initiation of a productive infection in hESC-derived neurons quiescently infected (GFP-negative) with VZV, as measured by ORF66GFP expression. NGF signaling has been shown to be required to maintain the quiescent state of HSV in the dissociated rodent sympathetic neuron model [24] [25], and can facilitate the maintenance of HSV latency in the murine ganglionic explant model [26] [27]. We therefore incubated persistently infected cultures in media lacking the three neurotrophin growth factors added to our neuronal cultures: NGF, BDNF and NT3 (18). In approximately 30% of GFP-negative wells (representing 50% of the original number of wells exposed to virus), individual GFP+ neurons were observed by 4 days after GF withdrawal, indicating ORF66kinase protein expression (S1B Table and Fig 4). Both single isolated and small foci of GFP+ neurons appeared, but GFP+ neurons did not increase in number with further incubation time, and most neurons in the culture had died by 5 days after GF removal. Similar results were obtained for 2, 4 and 7 wk quiescently-infected neurons (n = 3 each time point).

**Fig 5:**
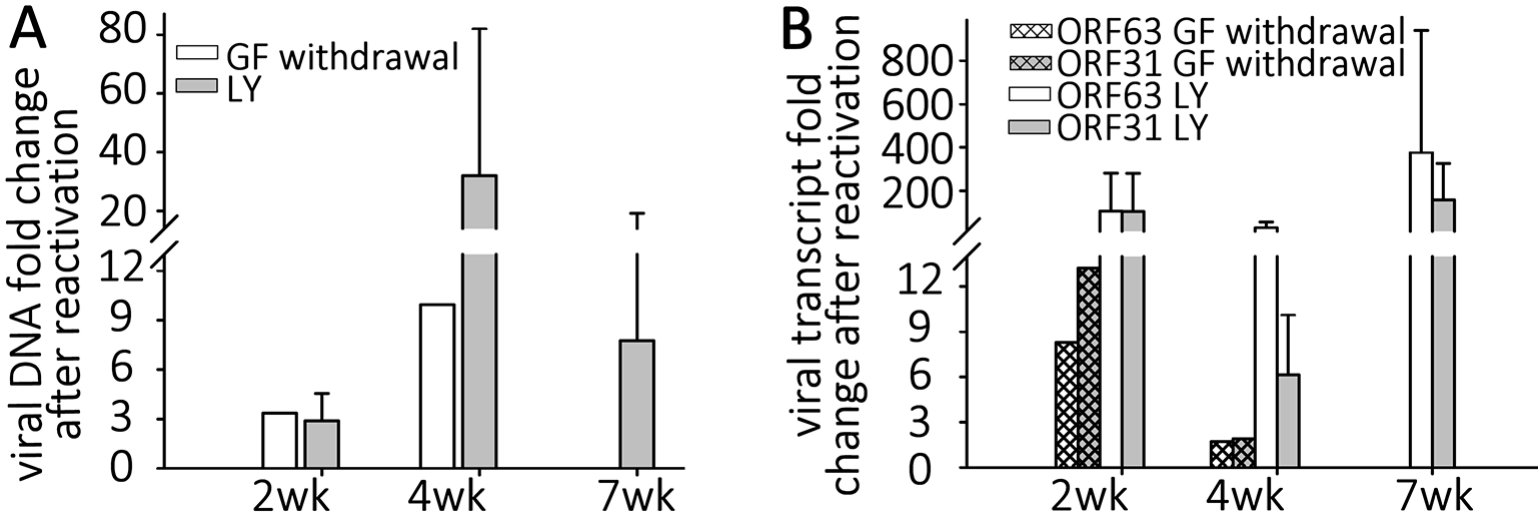
Reactivation stimuli increase the number of VZV genomes and transcripts in quiescently-infected human neurons. Wells of quiescently infected neurons were induced to reactivate VZV using growth factor withdrawal (GF n = 2 for each time point) or treatment with PI3K inhibitor LY294002 (LY) 2, 4, or 7 weeks (n = 5 for each time point) after infection. DNA and RNA were extracted from the wells and VZV genomes (A) and transcripts (B) of ORF63 and ORF31 were quantified. Both treatments increased the levels of both viral genomes and transcripts to varying degrees at all time points tested, indicating at least a partial reactivation of VZV. In all experiments reactivating VZV with LY, changes in nucleic acid levels measured were statistically significant.

The levels of VZV nucleic acids in wells undergoing GF withdrawal treatment were then determined and compared to those in parallel GFP-negative wells that continued to receive the three growth factors. At 2 wk post infection, GF withdrawal wells showed a modest increase in viral DNA and transcripts from ORF63 and ORF31 (Fig 5A and 5B). Cultures undergoing GF withdrawal at 4 weeks pi also showed elevated levels of viral DNA and both viral transcripts as compared to unstimulated controls (Fig 5A and 5B) (n = 2 at each time point). These data strongly suggest that GF withdrawal results in the initiation of reactivation events in hESC-neurons persistently infected with VZV. The relatively low numbers of GFP+ neurons could indicate a re-entry into a quiescent state, a failure of cells to support amplification of infectious virions due to cell changes resulting from growth factor withdrawal. The expression of GFP in a few neurons may also reflect a release of transcriptional repression in a non-productive “animation” event [28].

### Reactivation of VZV in neurons by PI3-K inhibition

NGF binding to its receptor TrkA results in cellular signaling through PI3-K, and this has been shown to be important in the maintenance of the quiescent state of HSV1, since treatment with the PI3-K inhibitor LY294002 (LY) results in HSV-1 reactivation [29]. Since GF withdrawal induced increases in VZV genomes and transcripts in persistently infected hESC derived neurons, we suspected that similar pathways might govern VZV latency and reactivation. Wells containing hESC-neurons quiescently infected (with VZV i.e. not containing GFP+ cells) for 2,4 and 7 wk (n = 5 for each time point) were therefore treated with LY, and observed for expression of GFP. Similar to the results obtained from GF withdrawal, LY treatment was observed to result in a few GFP+ cells or small foci appearing in about 1/3 of treated wells (S1C Table), consistent with a role for PI3K signaling contributing to VZV latency, The clusters of GFP+ neurons did not increase in size, but we were only able to follow the cultures for 3–4 days after LY treatment, due to its toxicity at this temperature (see below) resulting in the death of the neurons. qPCR analysis revealed that viral DNA and transcripts greatly increased with LY treatment, more so than observed after GF withdrawal (Fig 5A and 5B). Two week quiescently-infected, LY treated neurons contained more viral DNA, and increased transcription of ORF63 and ORF31 compared to wells with quiescently-infected neurons that were not treated. Similar studies of 4 wk and 7 wk quiescently infected neurons also revealed increases in viral DNA and transcripts. This indicates that PI-3K inhibition results in relaxation of VZV repression in hESC-derived neurons, viral gene transcription and the expression of viral protein. The presence of small foci of ORF66GFP positive cells in some wells strongly suggests full genome replication, viral transcription and virus production. We were also able to trigger reactivation using the type 1 histone deacetylases-inhibitor sodium butyrate (increases in VZV DNA and transcripts induced by sodium butyrate with and without LY are shown in S2A Fig).

FISH analyses of LY treated cultures were consistent with replication of VZV in the neurons. After 4 days of treatment with LY, 2.88% of the nuclei from treated wells were positive for viral DNA, but the number of FISH positive puncta in nuclei with a positive hybridization signal increased significantly. While 74% of the FISH positive nuclei of LY treated neurons contained one punctum, 6% two puncta, 8% 3 puncta, more than 12% of the cells contained over 3 puncta (Table 1). Although the hybridization events may not correspond to individual genomes, the increase in the number of hybridization signals is consistent with viral DNA replication.

### Reduced temperature enhances the effect of VZV reactivation stimuli upon neurons persistently infected by VZV, leading to spreading infection

In the preceding experiments, application of reactivation stimuli resulted in viral genome amplification and increased transcription in a fraction of wells of quiescently infected neurons, but the ORF66GFP expression events were limited to single cells or small foci. This contrasts the productive, spreading infection seen in hESC-derived neurons exposed to high MOI VZV (Fig 1). We and others (see discussion) have observed that VZV replication in non-neural cells is facilitated at temperatures 2–4°C lower than 37°C. Therefore, we combined LY treatment of persistently infected neurons with incubation at reduced temperature (34°C).

**Fig 6:**
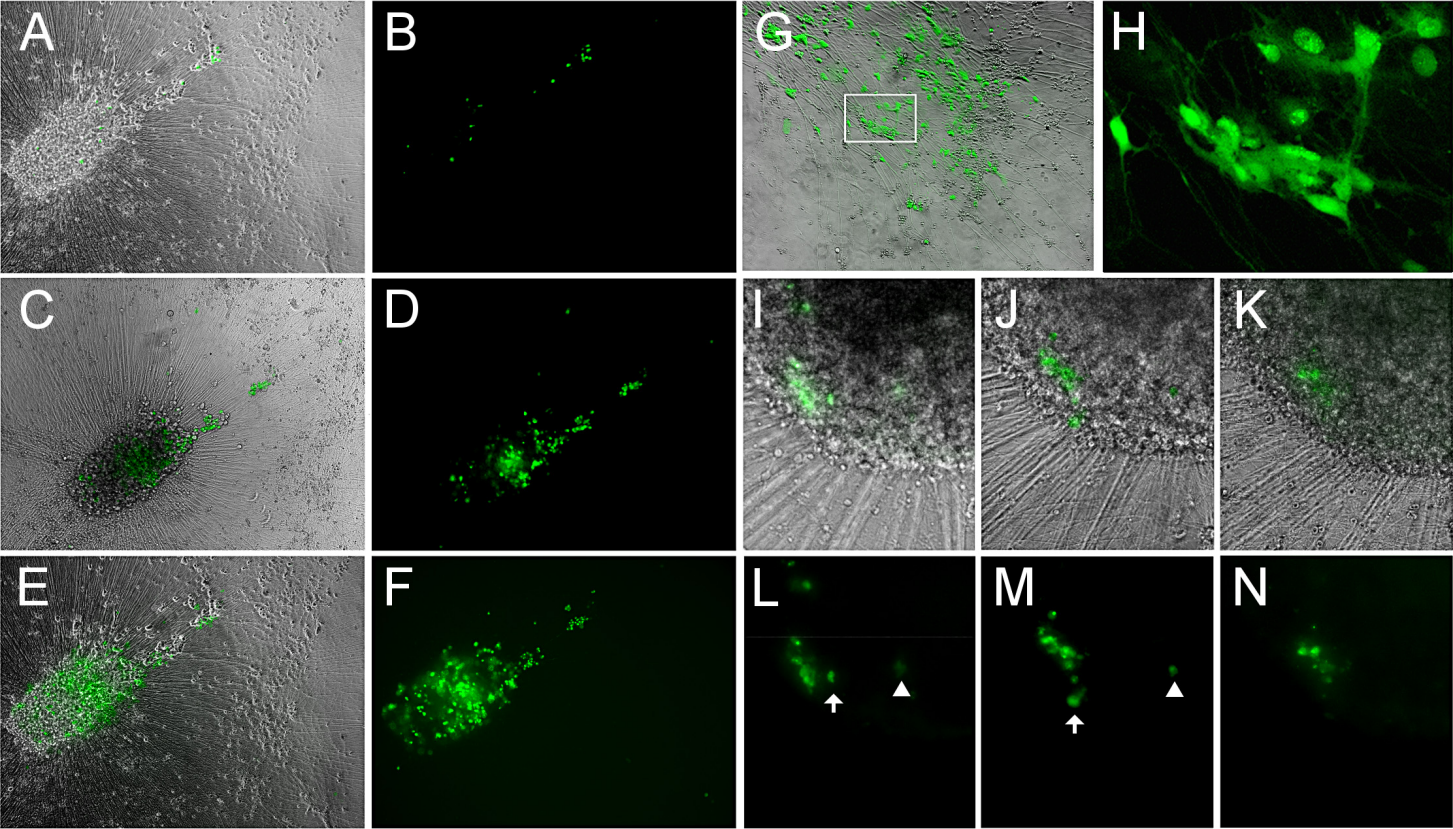
Reactivation of VZV in quiescently infected hESC-derived neurons by PI3K inhibition at 34°C results in a productive, spreading infection. (A-F) A microscopic field showing neurons expressing GFP after reactivation at 34°C for 2 (A,B), 6 (C,D) and 14 (E,F) days after initiation of treatment. GFP expression is first observed in individual neurons, and spreads over time in the same initial foci of expression. (G-H) Another experiment showing the results of LY-induced reactivation at 34°C. The area depicted by the box in G is presented at higher magnification in H, showing the diffuse filling of neurons with GFP at 34**°**C. (I-N) In another reactivation experiment at 34°C, a focus of GFP expression (I&L) initially spreads over 3 days (J&M), but then contracts over a period of 4 days (K&N). Scale bars = 100μm.

Multiwell plates containing neurons persistently-infected with VZV-66GFP one week after ACV withdrawal were transferred to 34°C, with half of the GFP- wells receiving LY, and half of the GFP- wells receiving only culture medium. Parallel cultures were maintained at 37°C. Persistently-infected cultures induced to reactivate at 34°C by LY(n = 3 independent experiments), showed a more rapid appearance of GFP+ neurons than observed at 37°C, with fluorescent neurons present by two days after induction. This is at least one day earlier than GFP+ neurons were observed with induction of reactivation at 37°C by LY or other stimuli. Furthermore, combined treatment of LY and low temperature resulted in the population of GFP+ neurons expanding for up to 14d (Fig 6A–6F). Enhanced VZV spread after induced reactivation at 34°C was observed in all (4/4) induced reactivation cultures.

We further established that infectious progeny virus was produced from the reactivation by trypsinizing neurons reactivated by LY at 34°C and re-seeding them onto ARPE19 cells. The ARPE cells were infected within one day as visualized by GFP expression, and rapidly developed syncytia and viral plaques.

However, even following incubation at 34°C with LY, not all individual GFP+ neurons produced a spreading infection, and some initially GFP+ neurons lost GFP expression (Fig 6H–6M). Lower temperature by itself was not an effective stimulus for VZV reactivation from persistent infection induced with ACV: one of four wells of neurons persistently-infected with VZV and then incubated at 34°C without LY treatment contained neurons expressing ORF66GFP. This suggests that the lower temperature primarily enhances virus spread rather than acting as a direct stimulus of reactivation.

### VZV transcripts transcribed from the short duplicated regions of the genome are enriched in silently-infected neurons

**Fig 7:**
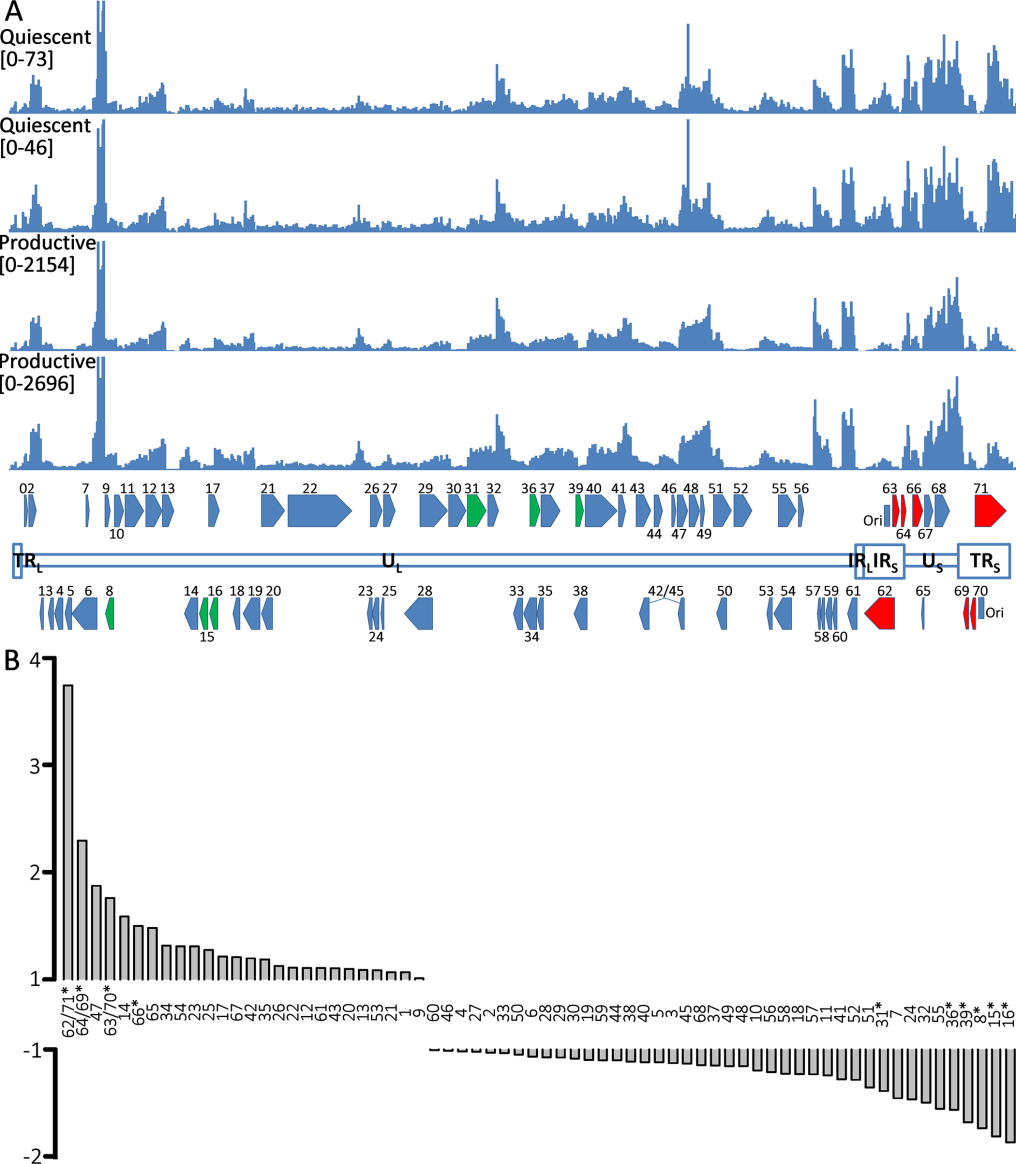
RNA expression in productively and quiescently-infected hESC-derived neurons. A) The upper two histograms show the counts of reads from RNASeq analysis of quiescently-infected neurons and the lower two those of productively infected neurons aligned to the annotated vOKA genome. Note the difference in the Y-axis scale between the sets of histograms depicting productive and quiescently infected cultures. In the annotated genome at the bottom of the Fig, ORFs depicted in red (increased) and green (reduced) are those displaying statically significant differences in enrichment between quiescent and productively infected neurons. B) Fold-changes between the relative expression of transcripts of VZV ORFs between quiescent and productively infected neurons. The duplicated genes of the short repeats region of the genome are enriched in quiescently-infected neurons. ORFs for which significant differences were detected are denoted by asterisks.

The ability to reactivate VZV genomes in hESC-derived neurons establishes the persistence observed in this *in vitro* model as a latency-like state. We therefore interrogated the VZV transcriptome in persistently-infected hESC-derived neurons and compared it to the VZV transcriptome of productively-infected neurons using RNA-seq (S2 Table contains a summary of RNASeq reads obtained). Alignment of transcript sequences with that of an annotated VARIVAX genome (that differs from the pOka genome used in these experiments by ~42bp [30]) (Fig 7A) revealed that transcripts from all genomic regions were expressed in both productively and quiescently infected hESC neurons, with levels of the viral transcripts between 20–50x higher in the productively infected cells, note the difference in Y-axis scale between the quiescent and productive alignments), as observed in the rt-PCR assays (Fig 2B). However, the level of transcription from different portions of the genome varied considerably within quiescent and lytic infected samples, and these differences were relatively consistent between the biological replicates. The S3 Table lists FKPM counts for the ORFs of the vOka annotated genome in descending order of expression. Notably, transcripts from the ORF57 gene, which is non-essential in MeWo cells [31] and has a PRV but not a HSV1 homolog, were expressed at the highest levels in both productively and quiescently infected neurons.

Striking differences in the relative levels of VZV transcripts was observed between quiescently and productively infected neurons for several genomic regions (Fig 7B). Specifically, transcripts for the duplicated regions of the genomes bounding the short unique genomic region—containing genes (62/71,64/69,63/70) were significantly enriched in quiescently compared to productively-infected neurons. Conversely, transcripts for ORF31, ORF36, ORF39, ORF8, and ORF15/16 were expressed at relatively lower levels in quiescently infected neurons.

### Persistent, silent infection and reactivation of hESC-derived neurons without the use of anti-viral drugs

**Fig 8:**
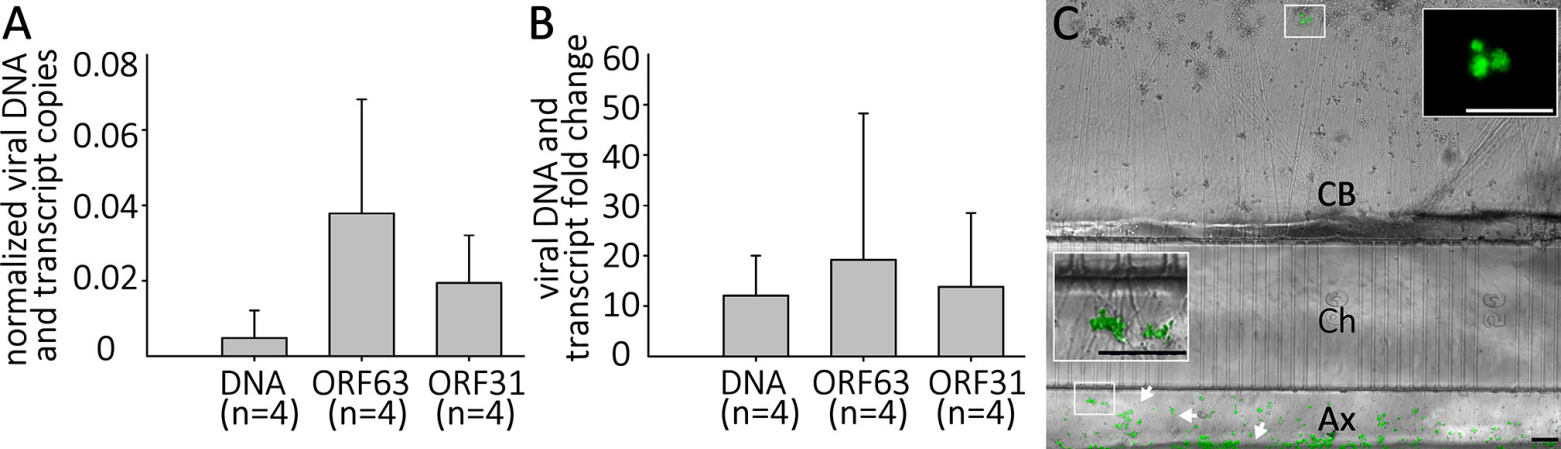
Quiescent infection and reactivation of VZV in hESC-derived neurons without the use of ACV. hESC-derived neurons were infected via their axons in compartmentalized microfluidic chambers as detailed in the methods. A) Quantification of VZV genomes and transcripts from ORF63 and ORF31 were quantified by digital qPCR from nuclei acids extracted from the cell-body compartment two weeks after infection. B) & C) Reactivation of VZV in axonally-infected neurons. (B) Reactivation of VZV in hESC-derived neurons by LY 2 weeks after quiescent axonal infection. Neurons infected as in A were treated for 4 days with LY at 37°C and DNA and RNA extracted. qPCR revealed an increase in VZV genomes and transcripts from ORF62 and ORF31. No GFP-positive neurons were observed under these reactivation conditions. (C) Reactivation of neurons axonally-infected by VZV at 34°C. A photomicrograph of a microfluidic chamber where the cell body compartment (CB) containing the somata of axonally infected neurons was treated for 4 days with LY at 34 degrees is shown. A cluster of neurons (upper box) expressing ORF66GFP as a result of the treatment (higher magnification image in upper inset). The axons in the axonal compartment (Ax) are coated by the GFP-fluorescent debris (lower box, higher magnification image in the lower inset) used to infect the axons. Ch = microfluidic channels connecting the cell body and axonal compartments. Scale Bars = 100μm.

In the human host, alphaherpesvirus latency is established in neurons without anti-viral treatment. In the course of a natural VZV infection, the latent state is established either via infection of axons in the skin and transport of the virus to the ganglia, or directly by T-cells that migrate to the ganglia [32],[33]. Studies of HSV-1 have demonstrated that latency is preferentially established when neurons are exposed to virus only at their axons [34]. We therefore investigated whether a latency-like state could be established after axonal infection with cell-free VZV in hESC-derived neurons using compartmented microfluidic chambers. At two wk pi, no axonally-infected cultures contained GFP+ neurons in the cell body compartments of the chambers, even though GFP+ virus from the infection clearly coated axonal processes in the axonal compartments (Fig 8). Despite the absence of GFP fluorescence reporting productive infection in the somal compartment, qPCR for VZV DNA and RNA revealed that the presence of both VZV genomes and transcripts (Fig 8). We conclude from these data that VZV entered the distal axons, was transported to the cell bodies and initiated a quiescent infection without expression of detectable levels of the ORF66 GFP reporter.

Parallel axonally-infected neuronal cultures induced for reactivation of VZV by LY treatment in the soma compartments showed increases in both VZV genomes and transcripts without the appearance of GFP+ neurons (Fig 8, n = 4). Strikingly, when such axonally-infected cultures were incubated 34°C in the presence of LY, GFP+ neurons appeared that in some cases subsequently formed foci of multiple infected cells (n = 2 independent experiments, total of 5 chambers,). These results demonstrate that a VZV persistent infection of hESC-derived neurons can be established following infection via their axons, and that this infection can be experimentally reactivated using the same stimuli used for reactivating persistent infections established using ACV.

## Discussion

Our operative definition of experimental latency is the maintenance of viral genomes without virus production for extended periods that can be reactivated into a state of productive virus infection that spreads to other cells. This definition distinguishes between latency and abortive, incomplete or partial infections, which may apply to latency models in non-permissive hosts or cell types unable to support a full, productive infection. The factors maintaining the latent state and the drivers of reactivation are of high importance, since by understanding them, we may eventually be able to target such processes for prevention of reactivation disease. Latency involves a program in which the normal lytic viral gene expression program leading to virion production is largely suppressed. However, recent studies suggest that latency may not only involve expression of specific transcripts or proteins that promote and maintain the latent state, but may also involve dynamic repression and de-repression of lytic genes without virus production. Some aspects of the lytic-latent-lytic switch have been elucidated from several rodent *in vitro* and *in vivo* models systems of HSV1 (reviewed in [35],[28],[24],[36]).

By contrast, little has been learned concerning the VZV latent state and factors leading to reactivation. Herpes zoster and the sequellae of post herpetic neuralgia and a host of neurological complications that may follow, remain serious worldwide health issues. By the definition of latency just outlined, a reactivatable model of the VZV latent state in human neurons has, until the present study, not been developed. The proposed models for VZV latency in mice [37] rats [38] and guinea pigs [12] have not been demonstrated to be reactivatable. In the more VZV-susceptible *in vitro* guinea pig enteric ganglion model, reactivation has only been shown by the overexpression of a viral transcriptional regulatory protein (ORF61), a stimulus that greatly influences the host cell transcriptional environment [39]. Long term quiescent infection of human fetal DRG transplanted to SCID mice [40] and neural precursors in suspension has been achieved [19], but experimental reactivation has not been reported. Thus, our hESC-derived neuron system, which has the ability to host a reactivatable VZV persistent state is novel and unique.

We established persistent, silent VZV infections in hESC-derived neurons using two methods. The first is similar to an established *in vitro* model for HSV1 latency, in which productive infection of rat cervical ganglia neurons is repressed using the DNA replication inhibitor ACV. Initially developed by Wilcox and Johnson [25],[41], this model has been recently refined using GFP-expressing HSV1 to elucidate factors affecting the lytic/latent switch [24]. The second approach uses axonal infection with cell-free VZV in compartmented cultures without the use of ACV. By contrast, axonal infection of hESC-derived neurons performed with cell associated VZV results in a lytic, spreading infection [16],[42],[43]. Axonal infection with (cell free) HSV1 has also been observed to lead primarily to a silent infection [34]. It has been proposed that axonal infection establishes quiescence due to the reduced delivery of tegument proteins to the nucleus that act to promote the lytic cycle [28]. We speculate that cell-free VZV infections lead to minimal tegument protein delivery, whereas infection with cell associated virus [16],[42] leads to more efficient virus entry into neurons and higher delivery of lytic-infection promoting tegument proteins. These may also be delivered via fusion of infecting cells and neurons in cell associated infections [44]. Since VZV replication can be reactivated in neurons persistently infected by either of two different methods, it seems unlikely that the use of ACV generates an artifactual model of persistence. Interestingly, after removal of ACV, HSV1 spontaneously reactivates from a fraction of ACV-established persistently infected cultures [29], while we have never observed VZV spontaneous reactivation after ACV removal in dozens of experiments over the course of years. This suggests that in our model for VZV latency, repression is maintained more tightly. This could reflect the situation in humans: while HSV may reactivate many times, VZV in most people does not show signs of reactivation and in those who do develop zoster, it usually only occurs once. A central unanswered question is how the different reactivation patterns of HSV and VZV are regulated. Now that a model of VZV persistence that can be reactivated has been established, this issue may be addressable experimentally.

The quiescent state of VZV infection of hESC-derived neurons shows several hallmarks of a latent state. First, VZV genomes are detectable in neuronal cultures for up to 7 weeks as shown by both qPCR and DNA *in situ* hybridization. Second, DNA FISH reveals small puncta of hybridization in 4–5% of the neurons, rather than large hybridization signals filling the nuclei seen in lytically VZV-infected neurons. These small foci are similar to those reported in FISH studies of quiescent HSV1 [23]. Third, low level transcription from two VZV genes was consistently detected in quiescently-infected neurons by qRT-PCR. The copy number of transcripts from ORF63 (the transcript most often detected in VZV latency studies in other systems [35] [1] was consistently higher than that from ORF31(gB), but both were far below than those in observed in lytic infections of the same cells. This observation was confirmed in RNASeq analyses (Fig 8 and S3 Table). It is not yet clear if these low levels of transcripts represent continuous transcription that occurs without detectable expression of the ORF66 GFP fluorescence, or represent sporadic transcriptional de-repression events reflecting VZV “animation.”

VZV in a fraction of the persistently infected neurons responded to stimuli by being reactivated in terms of expression of ORF66GFP reporter protein after prolonged periods of undetectable expression. ORF66GFP fluorescence was accompanied by increases of viral genomes and transcripts in the cultures, as well as the production of virus capable of infecting susceptible cells. However, most quiescently-infected neurons do not reactivate VZV in our cultures. About 4% of neurons contain viral genomes as detected by FISH, yet induced expression of ORF66 protein was largely restricted to few events in a culture of several thousand neurons. The lack of reactivation for some persistently infected neurons in response to experimental stimuli has also been observed for HSV1 [45] [46]. The observation that most quiescent genomes are not reactivatable is also seen in both *in vivo* and *in vitro* models for HSV1 and may apply to the other members of the herpesvirus family. It is possible that quiescently infected neurons consists of a mixed population where some are capable of responding to different reactivation stimuli, while others may not be able to support the complete reactivation process. Consequently, the low levels of reactivation seen here may reflect the number of quiescently-infected neurons able to support reactivation. Alternatively others may require different, unknown reactivation stimuli. This system may provide a platform to identify other VZV reactivation triggers.

We induced reactivation using stimuli that act through the PI3K pathway, growth factor removal and PI3K inhibition. This strongly suggests that the latency/reactivation switch of VZV is controlled to some extent by the same cellular pathways that affect this switch in HSV1 [29]. Reactivation of quiescent VZV was also achieved using the type 1 histone deacetylases inhibitor sodium butyrate, which favors a more permissive environment for transcription. The PI3K pathway also interacts with downstream events in the cell that can alter the state of chromatin, so it is possible that all three stimuli act through the same downstream effectors.

The dramatic contribution of reduced temperature to the outcome of *in vitro* reactivation we observed was not expected. This experimental condition was evaluated because of the long standing observation that VZV replicates more efficiently at lower temperature [47] and growth at 34°C to permits larger quantities of virus to be generated for virion purification studies [48]. While at 37°C, LY- induced reactivation events were mostly detected as single fluorescent cells or small foci, neurons reactivated at 34°C showed spread of infection to neighboring neurons, and the ability to infect non-neuronal cells when transferred to fresh cultures. However, it is not yet clear why reduced temperature enhances the spread of infection and the generation of infectious virus in our model. We speculate that higher temperature may favor the quiescent state for VZV, and the small plaques seen with reactivation events may represent re-entry of VZV into a quiescent state in secondarily infected neurons. Alternatively, this could reflect an aspect of *in vivo* VZV infection. In both varicella and zoster, the main site of lytic infection is the skin, which is at a slightly reduced temperature compared to the environment of the peripheral ganglia containing the reservoir of latent virus. The high core temperature may limit virus spread at the onset of latency as well as limit reactivation events. This is obviously a highly efficient process in humans, since the majority of people never suffer from a reactivation event leading to zoster. Such temperature related effects could be due to activity of viral enzymes, initiation of pathways at lower temperature or less efficient activity of innate antiviral responses. Regardless of the mechanism, this observation helps shed light on recent studies that reported the reactivation of VZV gene expression post-mortem [2], with the longer the period from death to assay, the higher the levels of VZV transcripts measured. The reactivation we observe *in vitro* at 34°C suggests that the reduced body temperature after death may have participated in a partial reactivation event of the virus and some transcription.

A recent study called for caution when interpreting experiments where herpesvirus reactivation is studied *in vitro*. It was found that stimuli causing apoptosis could activate a pathway in which herpesviruses from all three families began to replicate and transcribe their genomes and make proteins [49]. We do not believe that our experimental reactivation was due to apoptosis for two reasons. First, we obtained much stronger reactivation at low temperature, but there were much lower levels of cell death caused by the pharmacological agents: at 37°C LY or growth factor withdrawal-treated neurons died within 5 days, while at 34°C, they survived for two weeks. In addition, when we treated quiescently infected neuronal cultures with inducers of apoptosis TRAIL, doxorubicin and nocodazole, it did not result in the appearance GFP+ neurons or an increase in VZV transcripts or genomes using qPCR (S2B Fig).

This new model now permits detailed examination of gene expression during persistent infection. HSV1 encodes several species of non-translated RNA during latency both *in vivo* and *in vitro*, collectively termed LATs, that encode for multiple miRNAs and longer non-coding RNAs that may participate in the maintenance of latency [50] [51]. Non-coding RNAs, long or miRNAs, have not yet been found in latently-infected post-mortem ganglia or other models of VZV latency so far [52]. Investigations of transcripts and proteins expressed in VZV latent infection have been performed on cadaver and experimentally infected fetal ganglia in SCID mice which are potentially complicated by the effects of post-mortem changes [2], and/or presence of multiple cells types in intact ganglia [53]. Low levels of VZV transcripts have been detected in human ganglia using both of these models, but whether these are translated remains an open question. The transcripts most often associated with VZV latency in most models are those from the genomic region coding for ORF63. More recently guinea pig enteric ganglia [12] and human neural precursors in suspension [19] have been used to model VZV quiescence, but it is not yet clear how well these system parallels human primary neuronal latency.

The VZV transcriptome in a 95% pure population of human neurons reveals that transcripts from all of the VZV genomic regions are expressed in both quiescent and productive infection, with transcription in quiescently infected neurons at a level several orders of magnitude lower. A similar finding was reported in an RNASeq study of iPSC-derived neurons infected with vOKA at low MOI [6]. Interestingly, the ORFs with the highest levels of transcription in VZV infected neurons (the present study and [6]), fibroblasts [6] and keratinocytes [54] are ORFs 57, 49 and 9.

Strikingly, transcription of RNAs from the VZV genome mapping to the internal and terminal short repeats (IR_S_ and TR_S_) were significantly enriched in quiescently as compared to productively-infected neurons. This suggests that the repeat regions are more transcriptionally permissive when the genome is maintained in a repressed state. Transcription of ORF63, which lies in these genomic repeat regions, has been frequently reported in models of experimental VZV latency, but our results suggest that enhanced transcription during latency may occur from a larger region than just this ORF. Interestingly, the repeat regions of HSV1 were also reported to be preferentially transcribed in a fibroblast model of persistent non-productive HSV1 infection, where the expression of IE proteins is eliminated [55]. They postulated that one reason for the preferred transcription of the repeat regions was their higher G+C content, which may permit a more permissive transcriptional environment. We note that the repeat regions of VZV are also higher in G+C content as compared to the unique genomic regions. Elucidation of the mechanism and physiological implications of the favored transcription of the repeat regions requires further investigation using this model. An intriguing possibility is that this region of the genome transcribes as yet undiscovered non-coding RNAs involved in maintaining the latency state.

In conclusion, our development of a robust *in vitro* model for VZV latency that can be experimentally reactivated and be dynamically monitored will now permit the mechanistic and transcriptional events underlying VZV persistence and reactivation to be studied in greater detail.

## Materials and Methods

### Cells and viruses

The H9 (US National Stem Cell Bank (WA09) human embryonic stem cell line, human neonatal foreskin fibroblasts (HFF), PA6 (Riken cell Bank, Japan) and ARPE19 (human retinal pigment epithelium (ATCC#CRL-2302) were maintained as previously described [56]. Parent Oka-based VZV expressing GFP as fusion proteins to ORF66 [22] was described previously and propagated in ARPE19 cells. Cell-free virus (titers 2000–10,000 PFU/ml) and infected debris (titers approximately 10^5^ PFU/ml) preparation and concentration were performed as described previously [56].

### Neuronal differentiation

Neurons were differentiated from hESC-derived neural precursor-containing aggregates (neurospheres, NSP) generated by co-culturing hESC with the PA6 mouse stromal cell line as previously described [42]. Cultures were performed in 24-well culture plates, with 5–10 neurospheres seeded in each well. We estimate that each well was seed with between 50–100,000 neural precursors, based on performing digital PCR for GAPDH DNA on 10 uninfected neurospheres.

### Quiescent infection and reactivation of VZV in hESC-derived neurons

After a minimum of 10 days of terminal differentiation of neurons into cultures that contained extensive axonal outgrowth, cells were pretreated with acyclovir (ACV, 50 μM) for 24 h, and then incubated with recombinant cell-free VZV expressing GFP fused to the ORF66 protein kinase. Cultures were exposed to low multiplicity of infection VZV (MOI: approximately 0.001 based on an estimate of 50–100,000 cells/well) for two hours in the presence of ACV. After removal of virus, the neurons were maintained in the presence of ACV for 6 days. Media without ACV was then used to maintain the cultures up to 7 weeks post infection (pi), with changes twice/wk. Cultures were examined regularly over the incubation period for GFP expression microscopically. At 2, 4 and 7 weeks pi, wells that did not contain GFP+ neurons received one of two treatments to induce reactivation. Growth factor withdrawal was achieved by incubation in media lacking the three neurotrophic factors NGF, BDNF and NT3. Reactivation using PI3 kinase inhibition was performed using LY294002 hydrochloride (LY, 10 μM, Tocris, cat.# 1130). Where indicated in the text, LY-treated cultures were incubated at 34°C. The strategy for silent infection and reactivation is shown schematically in S1 Fig VZV reactivation was assessed by monitoring for GFP expression for 3–4 (37°C) or 7–14 (34°C) days and then photographic documentation. Total RNA and DNA were extracted for digital Taqman qPCR analysis or RNA for RNA-Seq analysis (see below).

### Infection and reactivation of VZV in axonally infected neurons

Microfluidic chambers with two compartments connected by microchannels (length, 450 μm; height, 3 μm; width, 10 μm) were prepared as previously described [42]. Briefly, hESC-derived neurospheres were plated adjacent to microchannels in one compartment (cell body compartment), and axonal extension induced with a growth factor gradient. Axons reaching the axonal compartment were infected with a VZV cell-free lysate we term the “debris” fraction [56] containing ~100,000 PFU/ml of infectious virus. A volume gradient of medium was established between the two compartments to prevent cell free VZV from accessing the soma compartment by diffusion [57] [16].

### Fluorescent DNA *in situ* (FISH) hybridization for detecting VZV genomes

A protocol obtained from the clinical cytogenetics laboratory of Meir Hospital, Kfar Sabba, Israel was used for DNA *in situ* hybridization on nuclei isolated from the neurons. A VZV DNA probe labeled with DIG was generated and hybrization detected by indirect immunofluorescence for DIG. 1684 nuclei were examined, pooled from 3 independent experiments Details of the method are in S1 Methods.

### Quantitative PCR for detecting VZV genomes and transcripts in human neurons

DNA and total RNA were extracted simultaneously using Tri-Reagent (Sigma). Total RNA was reverse-transcribed using an oligo dT primer and M-MLV reverse transcriptase. Gene specific DNA probes for viral genes ORF31 and ORF 63 and human GAPDH were used for quantification with a digital PCR in duplicate samples. Copy number of viral DNA and transcripts were normalized using human GAPDH. Others have reported GAPDH expression is not affected significantly by VZV infection and is therefore a good standard for normalization of rt-PCR results [58]. Sequences of primers and probes and additional details are provided in the S1 Methods and in the S4 Table.

### RNA-seq analysis of the VZV transcriptome in silently and productively-infected neurons

Cultures of human neurons were infected, and 1 week after ACV withdrawal RNA was extracted from cultures not containing any GFP+ cells or from neurons productively infected with high-MOI cell-free VZV. Total RNA (less ribosomal RNA) was labeled with the TruSeq Stranded Total RNA LT Sample Prep Kit (with Ribo-Zero Gold) (#RS-122-2301) and run on an Illumina HiSeq 2500 sequencer at the Crown Institute for Genomics at the Weizmann Institute, Rehovot, Israel. Analysis was performed using bioinformatics tools as described in the S1 Methods.

### Microscopy

Live cultures were monitored with Olympus IX70 or IX81 microscopes and photographed with digital cameras. Images were enhanced using ImageJ and Paint Shop Pro software with all changes in the images (i.e., contrast, brightness, gamma, and sharpening) made evenly across the entire field. No features were removed or added digitally.

## Supporting Information

S1 Fig Schematic representation of hESC-neuron based model for VZV latency and reactivation. A) hESC- derived neuronal cultures in multiwell plates were infected with low PFU of VZV-GFP66 and incubated in the presence of acyclovir for 6 days, followed by incubation of the cultures in media without ACV. Approximately half of the wells contained some neurons that were GFP+, while the other half did not contain detectable GFP fluorescence. After 2–7 weeks further incubation, cultures received a reactivation stimulus, as detailed in the text. Neurons expressing ORF66-GFP fluorescence appeared in approximately 30% of treated wells incubated at 37°C within 2–4 days.
(DOCX)

S2 Fig S2A Fig depicts graphically the increase in the number of VZV genomes and transcripts in quiescently-infected human neurons after treatment with sodium butyrate or a combination of sodium butyrate (NaB) and LY. Quantitation of genomes and transcripts was by digital PCR. Both treatments increased the levels of both viral genomes and transcripts to varying degrees at all time points tested indicating at least a partial reactivation of VZV. The data shown for the NaB experiments are averages of 3 independent experiments at each time point. The data for the combination treatments are averages of: 2 wk, 10 experiments, 4 wk, 5 experiments and 7 wk, 8 experiments.S2B Fig is a graph showing the results of treatment of cultures of hESC-derived neurons quiescently-infected with VZV with inducers of apoptosis. The treatments caused the cells to die within 4 days, but did not induce the replication of VZV genomes or transcription of VZV genes. The graph shows the average of results from 2 independent experiments.
(DOCX)

S1 Table Table A provides data from 7 experiments showing the number of wells treated with low MOI VZV and ACV that were GFP+ and GFP- one week after ACV withdrawal. Table B provides data indicating how many initially GFP- wells were induced to contain ORF66GFP+ neurons after withdrawal of growth factors from the medium at 37°C. Table C provides data indicating how many initially GFP- wells were induced to contain ORF66GFP+ neurons after addition of a PI3K-inhibitor, LY, to the medium at 37°C.
(DOCX)

S2 Table Summary of RNASeq reads. Two biological repetitions of productive and quiescently infected neurons were analyzed and aligned to a vOka annotated genome. The number of total reads was similar for all samples. The VZV-specific reads were approximately 20–50x higher in productively infected compared to quiescently infected neurons. The RNASeq reads mapping to the invert repeat regions were done randomly and equally partitioned between the two gene copies.
(DOCX)

S3 Table Quantification of VZV ORF transcripts in quiescently and productively infected neurons. The data is arranged in descending order of expression. Transcripts with significant differences in enrichment between the two groups are shaded in yellow. Note that the three most highly expressed transcripts in both types of infection are the same.
(DOCX)

S4 Table Primers and probes used for qPCR quantification of VZV genomes or transcripts. (DOCX)

S1 Methods (DOCX)
