# Supplementary material for: An In Vitro Model of Latency and Reactivation of Varicella Zoster Virus in Human Stem Cell-Derived Neurons
Source: PLoS Pathog. 2015 Jun 4;11(6):e1004885. doi: 10.1371/journal.ppat.1004885 (PMC4456082; doi:10.1371/journal.ppat.1004885)

**S1 Fig Supplementary Figure1**

Supplementary Figure 1 **Schematic representation of hESC-neuron based model for VZV latency and reactivation.** A) hESC- derived neuronal cultures in multiwell plates were infected with low PFU of VZV-GFP66 and incubated in the presence of acyclovir for 6 days, followed by incubation of the cultures in media without ACV. Approximately half of the wells contained some neurons that were GFP+, while the other half did not contain detectable GFP fluorescence. After 2-7 weeks further incubation, cultures received a reactivation stimulus, as detailed in the text. Neurons expressing ORF66-GFP fluorescence appeared in approximately 30% of treated wells incubated at 37^0^C within 2-4 days.


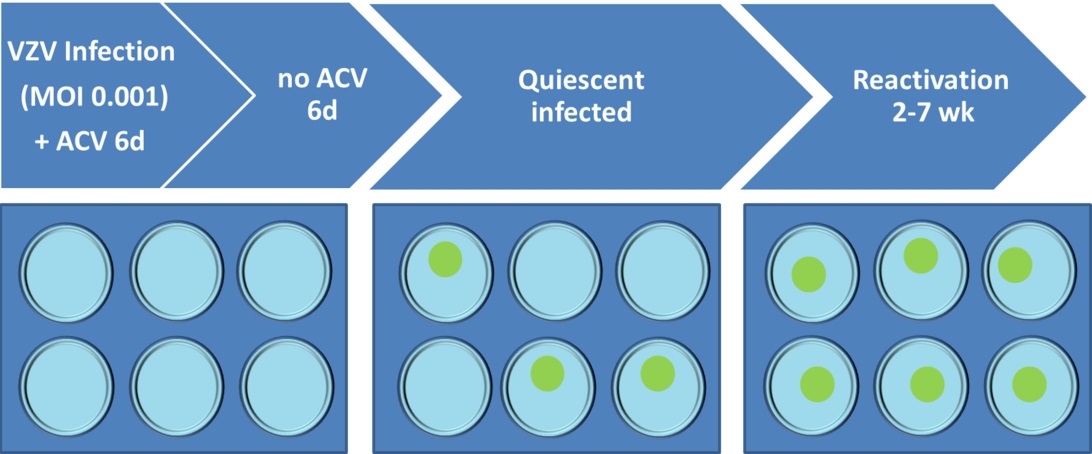

Supplement: S1 Fig — A) hESC- derived neuronal cultures in multiwell plates were infected with low PFU of VZV-GFP66 and incubated in the presence of acyclovir for 6 days, followed by incubation of the cultures in media without ACV. Approximately half of the wells contained some neurons that were GFP+, while the other half did not contain detectable GFP fluorescence. After 2–7 weeks further incubation, cultures received a reactivation stimulus, as detailed in the text. Neurons expressing ORF66-GFP fluorescence appeared in approximately 30% of treated wells incubated at 37°C within 2–4 days. (DOCX) [file ppat.1004885.s001.docx]
