# Supplementary material for: An In Vitro Model of Latency and Reactivation of Varicella Zoster Virus in Human Stem Cell-Derived Neurons
Source: PLoS Pathog. 2015 Jun 4;11(6):e1004885. doi: 10.1371/journal.ppat.1004885 (PMC4456082; doi:10.1371/journal.ppat.1004885)
Supplement: S2 Fig — Quantitation of genomes and transcripts was by digital PCR. Both treatments increased the levels of both viral genomes and transcripts to varying degrees at all time points tested indicating at least a partial reactivation of VZV. The data shown for the NaB experiments are averages of 3 independent experiments at each time point. The data for the combination treatments are averages of: 2 wk, 10 experiments, 4 wk, 5 experiments and 7 wk, 8 experiments.S2B Fig is a graph showing the results of treatment of cultures of hESC-derived neurons quiescently-infected with VZV with inducers of apoptosis. The treatments caused the cells to die within 4 days, but did not induce the replication of VZV genomes or transcription of VZV genes. The graph shows the average of results from 2 independent experiments. (DOCX) [file ppat.1004885.s002.docx]

**S2 Figs Supplementary figures S2A and S2B**

S2A Fig

Increase in the number of VZV genomes and transcripts in quiescently-infected human neurons after treatment with sodium butyrate or a combination of sodium butyrate (NaB) and LY. Quantitation of genomes and transcripts was by digital PCR.

Both treatments increased the levels of both viral genomes and transcripts to varying degrees at all time points tested indicating at least a partial reactivation of VZV. The date shown for the NaB experiments are averages of 3 independent experiments at each time point. The data for the combination treatments are averages of: 2 wk, 10 experiments, 4 wk, 5 experiments and 7 wk, 8 experiments.


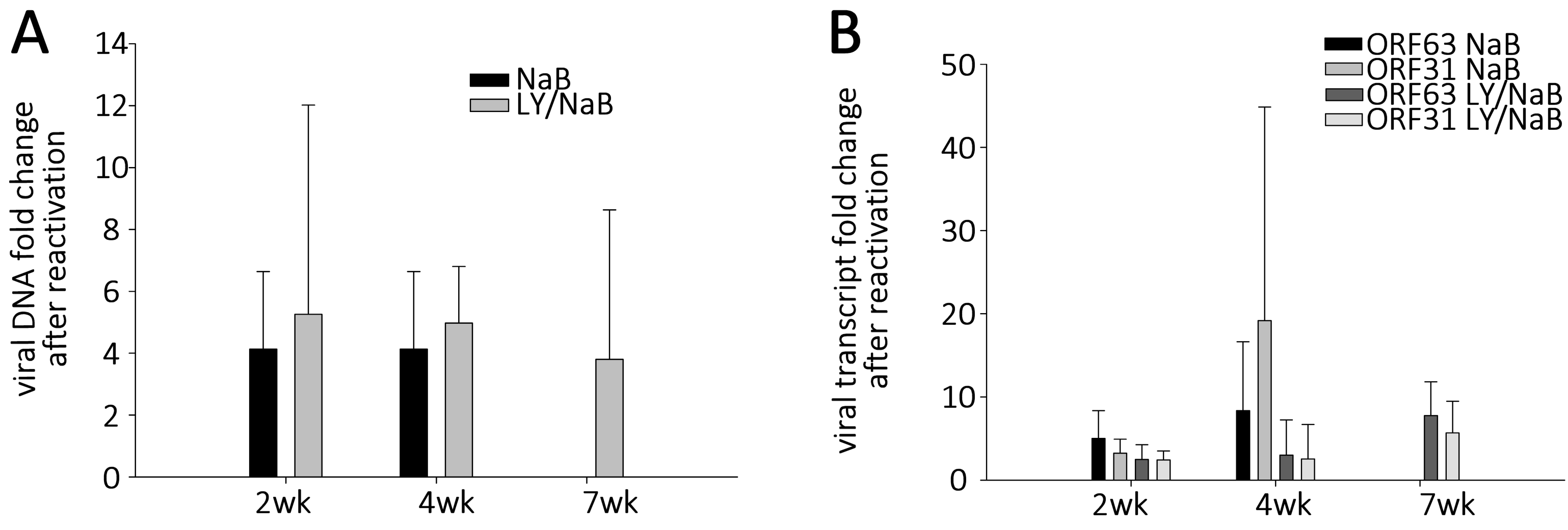


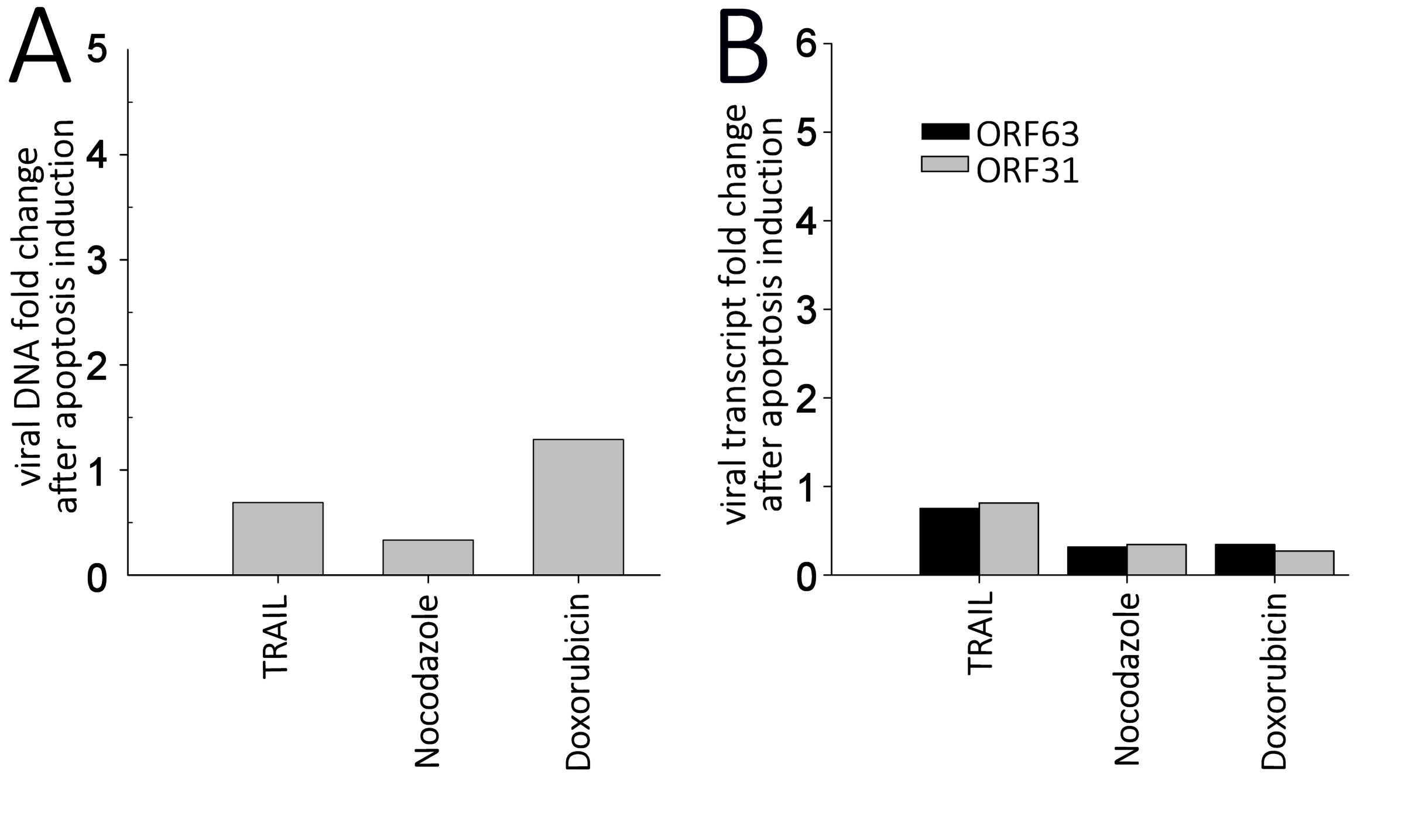
S2B Fig

Treatment of cultures of hESC-derived neurons quiescently-infected with VZV with inducers of apoptosis. The treatments caused the cells to die within 4 days, but did not induce the replication of VZV genomes or transcription of VZV genes. The graph shows the average of results from 2 independent experiments.
