# Supplementary material for: An In Vitro Model of Latency and Reactivation of Varicella Zoster Virus in Human Stem Cell-Derived Neurons
Source: PLoS Pathog. 2015 Jun 4;11(6):e1004885. doi: 10.1371/journal.ppat.1004885 (PMC4456082; doi:10.1371/journal.ppat.1004885)
Supplement: S1 Table — Table B provides data indicating how many initially GFP- wells were induced to contain ORF66GFP+ neurons after withdrawal of growth factors from the medium at 37°C. Table C provides data indicating how many initially GFP- wells were induced to contain ORF66GFP+ neurons after addition of a PI3K-inhibitor, LY, to the medium at 37°C. (DOCX) [file ppat.1004885.s003.docx]

**S1 Supplementary Tables A-C**

**Table A**

Numbers of wells containing GFP+ and GFP- neurons 2wk after exposure to low MOI cell-free VZV and one week after withdrawal of acyclovir from the medium

| Experiment | Wells **containing** GFP expressing neurons | Wells **without** GFP expressing neurons  (quiescent infection) | Percentage of wells with quiescently infected neurons |
| --- | --- | --- | --- |
| 1 | 3 | 3 | 50% |
| 2 | 2 | 3 | 60% |
| 3 | 4 | 12 | 75% |
| 4 | 3 | 3 | 50% |
| 5 | 10 | 6 | 38% |
| 6 | 4 | 8 | 67% |
| 7 | 10 | 8 | 44% |

**Table B**

Numbers of wells containing neurons quiescently-infected with VZV (GFP-)that responded to growth factor withdrawal at 37^0^C by expression of ORF66GFP (reactivation)

| Number of weeks post infection | Wells **containing** neurons with reactivated VZV | Wells **lacking** neurons with reactivated VZV | Percentage of wells responding to GF-withdrawal by reactivation |
| --- | --- | --- | --- |
| 2 | 3 | 1 | 33% |
| 2 | 3 | 1 | 33% |
| 4 | 4 | 1 | 25% |
| 4 | 3 | 1 | 33% |
| 7 | 2 | 0 | 0% |

**Table C**

Numbers of wells containing neurons quiescently-infected with VZV (GFP-)that responded to LY treatment at 37^0^C by expression of ORF66GFP (reactivation)

| Number of weeks post infection | Wells **containing** neurons with reactivated VZV | Wells **lacking** neurons with reactivated VZV | Percentage of wells responding to LY addition by reactivation |
| --- | --- | --- | --- |
| 2 | 3 | 1 | 33% |
| 2 | 3 | 1 | 33% |
| 2 | 3 | 0 | 0 |
| 2 | 4 | 1 | 25% |
| 2 | 3 | 2 | 67% |
| 4 | 3 | 0 | 0 |
| 4 | 2 | 2 | 100% |
| 4 | 3 | 1 | 33% |
| 7 | 2 | 1 | 50% |
| 7 | 3 | 0 | 0 |
