# Supplementary material for: An In Vitro Model of Latency and Reactivation of Varicella Zoster Virus in Human Stem Cell-Derived Neurons
Source: PLoS Pathog. 2015 Jun 4;11(6):e1004885. doi: 10.1371/journal.ppat.1004885 (PMC4456082; doi:10.1371/journal.ppat.1004885)
Supplement: S2 Table — Two biological repetitions of productive and quiescently infected neurons were analyzed and aligned to a vOka annotated genome. The number of total reads was similar for all samples. The VZV-specific reads were approximately 20–50x higher in productively infected compared to quiescently infected neurons. The RNASeq reads mapping to the invert repeat regions were done randomly and equally partitioned between the two gene copies. (DOCX) [file ppat.1004885.s004.docx]

**S2 Supplementary Table**

**Summary of RNASeq reads**. Two biological repetitions of productive and quiescently infected neurons were analyzed and aligned to a vOka annotated genome. The number of total reads was similar for all samples. The VZV-specific reads were approximately 20-50x higher in productively infected compared to quiescently infected neurons.

|  | total reads VZV+neurons | VZV-reads | % reads aligned to VZV genome |
| --- | --- | --- | --- |
| productive1 | 55468091 | 622840 | 1.12 |
| productive2 | 45000171 | 822933 | 1.83 |
| quiescent1 | 46317429 | 21018 | 0.05 |
| quiescent2 | 51139610 | 17219 | 0.03 |
