# Supplementary material for: An In Vitro Model of Latency and Reactivation of Varicella Zoster Virus in Human Stem Cell-Derived Neurons
Source: PLoS Pathog. 2015 Jun 4;11(6):e1004885. doi: 10.1371/journal.ppat.1004885 (PMC4456082; doi:10.1371/journal.ppat.1004885)
Supplement: S3 Table — The data is arranged in descending order of expression. Transcripts with significant differences in enrichment between the two groups are shaded in yellow. Note that the three most highly expressed transcripts in both types of infection are the same. (DOCX) [file ppat.1004885.s005.docx]

**S3 Supplementary Table**

Quantification of VZV ORF transcripts in quiescently and productively infected neurons. The data is arranged in descending order of expression. Transcripts with significant differences in enrichment between the two groups are shaded in yellow. Note that the three most highly expressed transcripts in both types of infection are the same.

| latent | | | productive | | |
| --- | --- | --- | --- | --- | --- |
| ORFs | latent  (FPKM) | % of total  viral RNA | ORFs | productive  (FPKM) | % of total  viral RNA |
| ORF57 | 136928 | 12.68 | ORF57 | 169287 | 15.46 |
| ORF49 | 106165 | 9.83 | ORF49 | 122750 | 11.21 |
| ORF9 | 87308 | 8.08 | ORF9 | 86372.4 | 7.89 |
| ORF66 | 42896.6 | 3.97 | ORF68 | 46069 | 4.21 |
| ORF67 | 42894.6 | 3.97 | ORF67 | 35524.8 | 3.24 |
| ORF68 | 40066.4 | 3.71 | ORF61 | 33910.7 | 3.10 |
| ORF47 | 39509.7 | 3.66 | ORF58 | 33729.5 | 3.08 |
| ORF61 | 37523.8 | 3.47 | ORF41 | 28922.8 | 2.64 |
| ORF58 | 27431.3 | 2.54 | ORF66 | 28622.3 | 2.61 |
| ORF63/70 | 26914.15 | 2.49 | ORF4 | 26090 | 2.38 |
| ORF4 | 25581.4 | 2.37 | ORF48 | 24428.5 | 2.23 |
| ORF64/69 | 25549.1 | 2.37 | ORF13 | 22824.6 | 2.08 |
| ORF13 | 24800.5 | 2.30 | ORF33 | 22163.4 | 2.02 |
| ORF41 | 22560.3 | 2.09 | ORF47 | 21097.7 | 1.93 |
| ORF33 | 21387.9 | 1.98 | ORF59 | 18139 | 1.66 |
| ORF48 | 21108 | 1.95 | ORF32 | 17883.1 | 1.63 |
| ORF62/71 | 17394.13 | 1.61 | ORF46 | 17130.5 | 1.56 |
| ORF60 | 16963.1 | 1.57 | ORF60 | 17099.6 | 1.56 |
| ORF46 | 16887.2 | 1.56 | ORF63/70 | 15055.88 | 1.37 |
| ORF59 | 16450.1 | 1.52 | ORF24 | 14941.4 | 1.36 |
| ORF50 | 14192.3 | 1.31 | ORF50 | 14896.7 | 1.36 |
| ORF20 | 13750.8 | 1.27 | ORF20 | 12544.8 | 1.15 |
| ORF53 | 12904 | 1.19 | ORF40 | 12266 | 1.12 |
| ORF12 | 11964.3 | 1.11 | ORF18 | 12024.6 | 1.10 |
| ORF32 | 11933.3 | 1.10 | ORF53 | 11906.5 | 1.09 |
| ORF40 | 10952.5 | 1.01 | ORF3 | 11725.8 | 1.07 |
| ORF38 | 10475.9 | 0.97 | ORF38 | 11678 | 1.07 |
| ORF3 | 10360.6 | 0.96 | ORF31 | 11466.3 | 1.05 |
| ORF42 | 10253 | 0.95 | ORF37 | 10902.2 | 1.00 |
| ORF24 | 10178.2 | 0.94 | ORF12 | 10807 | 0.99 |
| ORF18 | 9774.74 | 0.90 | ORF64/69 | 10678.385 | 0.98 |
| ORF37 | 9459.09 | 0.88 | ORF10 | 10308.9 | 0.94 |
| ORF10 | 8595.43 | 0.80 | ORF15 | 8986.51 | 0.82 |
| ORF65 | 8431.5 | 0.78 | ORF44 | 8859.84 | 0.81 |
| ORF31 | 8233.88 | 0.76 | ORF42 | 8577.9 | 0.78 |
| ORF54 | 8031.52 | 0.74 | ORF36 | 8436.61 | 0.77 |
| ORF44 | 8026.63 | 0.74 | ORF19 | 6802.98 | 0.62 |
| ORF34 | 7937.17 | 0.73 | ORF7 | 6408.79 | 0.59 |
| ORF25 | 7801.46 | 0.72 | ORF54 | 6132.66 | 0.56 |
| ORF23 | 7556.43 | 0.70 | ORF25 | 6120.67 | 0.56 |
| ORF19 | 6180.74 | 0.57 | ORF34 | 6040.06 | 0.55 |
| ORF27 | 5677.09 | 0.53 | ORF5 | 5959.26 | 0.54 |
| ORF29 | 5424.22 | 0.50 | ORF29 | 5857.29 | 0.53 |
| ORF36 | 5390.69 | 0.50 | ORF27 | 5802.65 | 0.53 |
| ORF5 | 5309.96 | 0.49 | ORF23 | 5782.61 | 0.53 |
| ORF15 | 4957.73 | 0.46 | ORF65 | 5699.99 | 0.52 |
| ORF7 | 4405.84 | 0.41 | ORF45 | 4969.59 | 0.45 |
| ORF45 | 4388.93 | 0.41 | ORF62/71 | 4588.08 | 0.42 |
| ORF43 | 3696.33 | 0.34 | ORF11 | 4510.25 | 0.41 |
| ORF11 | 3624.32 | 0.34 | ORF16 | 4118.71 | 0.38 |
| ORF21 | 3592.05 | 0.33 | ORF8 | 3774.51 | 0.34 |
| ORF17 | 3215.91 | 0.30 | ORF21 | 3366.04 | 0.31 |
| ORF22 | 3076.49 | 0.28 | ORF43 | 3346.21 | 0.31 |
| ORF28 | 2812.71 | 0.26 | ORF28 | 3026.33 | 0.28 |
| ORF35 | 2551.99 | 0.24 | ORF22 | 2771.34 | 0.25 |
| ORF2 | 2271.74 | 0.21 | ORF39 | 2764.44 | 0.25 |
| ORF16 | 2203.46 | 0.20 | ORF17 | 2649.2 | 0.24 |
| ORF8 | 2173.25 | 0.20 | ORF55 | 2563.96 | 0.23 |
| ORF26 | 1964.97 | 0.18 | ORF2 | 2345.87 | 0.21 |
| ORF30 | 1914.99 | 0.18 | ORF35 | 2152.89 | 0.20 |
| ORF6 | 1735.81 | 0.16 | ORF30 | 2087.14 | 0.19 |
| ORF55 | 1648.83 | 0.15 | ORF6 | 1855.39 | 0.17 |
| ORF39 | 1643.84 | 0.15 | ORF26 | 1749.48 | 0.16 |
| ORF52 | 1124.07 | 0.10 | ORF52 | 1447.58 | 0.13 |
| ORF56 | 811.906 | 0.08 | ORF56 | 985.367 | 0.09 |
| ORF51 | 595.824 | 0.06 | ORF51 | 808.338 | 0.07 |
| ORF1 | 472.957 | 0.04 | ORF1 | 443.622 | 0.04 |
