# Supplementary material for: An In Vitro Model of Latency and Reactivation of Varicella Zoster Virus in Human Stem Cell-Derived Neurons
Source: PLoS Pathog. 2015 Jun 4;11(6):e1004885. doi: 10.1371/journal.ppat.1004885 (PMC4456082; doi:10.1371/journal.ppat.1004885)
Supplement: S4 Table — (DOCX) [file ppat.1004885.s006.docx]

**S4 Supplementary Table**

**Primers and probes used for qPCR quantification of VZV genomes or transcripts**

| Primer or probe | Sequence |
| --- | --- |
| VZV-ORF31 primer (forward) | 5'-GATGGTGCATACAGAGAACATTCC-3' |
| VZV-ORF31 primer (reverse) | 5'-CCGTTAAATGAGGCGTGACTAA-3' |
| VZV-ORF31 probe | 5'-(FAM)-TCCGCGCTGCAGGTTCCAGTAAT-(BHQ)-3' |
| VZV-ORF63 primer (forward) | 5'-ATTGAGGCGCCGAATGTTC-3' |
| VZV-ORF63 primer (reverse) | 5'-CTTCACCACCATCATCAGATACG-3' |
| VZV-ORF63 probe | 5'-(HEX)-TTTGCATAGGAGCGCACTGGAATGTG-(BHQ)-3' |
| Human GAPDH primer (forward) | 5'-CACATGGCCTCCAAGGAGTAA-3' |
| Human GAPDH primer (reverse) | 5'-TGAGGGTCTCTCTCTTCCTCTTGT-3' |
| Human GAPDH probe | 5'-(FAM)CTGGACCACCAGCCCCAGCAAG(BHQ)-3' |

The VZV sequences were taken from Pevenstein et al. 1999. J. Virol. 73:10514–10518, and the GAPDH sequences from Cohrs and Gilden 2007 J. Virol. 81:2950–2956.
