# Supplementary material for: An In Vitro Model of Latency and Reactivation of Varicella Zoster Virus in Human Stem Cell-Derived Neurons
Source: PLoS Pathog. 2015 Jun 4;11(6):e1004885. doi: 10.1371/journal.ppat.1004885 (PMC4456082; doi:10.1371/journal.ppat.1004885)
Supplement: S1 Methods — (DOCX) [file ppat.1004885.s007.docx]

**S1 Methods Supplementary methods**

**Fluorescent DNA *in situ* (FISH) hybridization for detecting VZV genomes**

The polylysine and laminin coating used in neuron cultures gave rise to considerable non-specific binding with fluorescent DNA in-situ hybridization (FISH) probes. We therefore modified a protocol used at the clinical cytogenetics laboratory of Meir Hospital, Kfar Sabba, Israel, in which FISH was performed on nuclei isolated from the neurons. Neuronal cultures were trypsinized, and the cells washed and centrifuged for 5’ at 1000g, and then incubated in 40 mM KCl at 37°C for 2h. After centrifugation for 5min at 1000 g, the pellet was subjected to three rounds of re-suspension in fixative (3:1 methanol:acetic acid), re-centrifuged and the supernatant was discarded. The nuclei were then placed on 13mm diameter round glass coverslips in a 24 well dish and centrifuged for 10’ at 1000g. Coverslips were washed for 10’ in SSCX2 (0.15 M NaCl plus 0.015 M sodium citrate) at 37^0^C and fixed with 4% buffered paraformaldehyde for 15’ at room temperature. Sequential dehydration of the coverslips in ethanol was performed (70%, 90% and 100%) for 2’ in each solution, followed by air drying.

FISH used a hybridization mix (50% formamide, 10% dextran sulfate, 1× SSC, 1× Denhardt's solution, 0.5 mg herring sperm DNA) contained 1 μl VZV DNA probe (HindIII digestion fragment of VZV genomic DNA) labeled with a DIG DNA-labeling kit (Roche Diagnostics, Penzberg, Germany). Probe was denatured at 98^o^C, and nuclei on the coverslips were denatured in the presence of 20 μl of hybridization mix at 85^o^C and incubated at 37°C in a humid chamber for 16 h. After hybridization, coverslips were washed three times for 5’ in 2×SSC at 37^o^C and 3x for 5’ in 0.1×SSC at 60^o^C and then blocked with 3% BSA in SSCX4 for 30’. Coverslips were incubated with anti-DIG monoclonal antibody (Jackson Immunochemicals, cat.#) for 1h at RT. A secondary antibody coupled to AlexaFluor 594 (Jackson) was applied for 30’ at RT, followed by removal of unbound excess by washing. After staining, coverslips were mounted on microscope slides in 90% glycerol–10% PBS with 1% n-propyl-gallate and sealed.

**Quantitative digital PCR for detecting VZV genomes and transcripts in human neurons**

DNA and total RNA were extracted simultaneously using TRI Reagent reagent (Sigma, cat.#T9424) according to the manufacturer’s protocol. Unamplified total RNA was reverse-transcribed using an oligo dT primer and M-MLV reverse transcriptase (Promega, cat.# M1705). DNA samples were cleaved into smaller fragments for dd-PCR reaction as per the instrument’s manual using HindIII digestion. The yield of RNA was about 5 μg of total RNA and 5 μg of DNA from each 3 wells of infected neuronal cultures. All PCR reactions were performed in duplicates. 20 μl of real-time PCR reactions were prepared with 2x ddPCR Supermix for probes (Bio-Rad, USA) and gene specific DNA probes for viral genes ORF31 and ORF 63 and human GAPDH. (Supplementary Table 1). Droplets were prepared using the QX 100 droplet generator (Bio-Rad, USA) following the manufacturer's instructions. Standard PCR was performed for 40 cycles and the reactions were analyzed on the QX 100 Droplet Reader. All data were further analyzed using the QuantaSoft program (v 1.6.6; Bio-Rad). Copy numbers of viral DNA and transcripts were normalized using human GAPDH.

**Analysis of RNASeq data**

4 samples were run on one lane of an Illumina HiSeq 2500 sequencer in high output mode, and yielded 45 to 55 million total reads per sample. Data was aligned to the annotated vOKA genome (GenBank: DQ008355.1) using the program Bowtie2[1]. For comparison of expression levels between samples the program cuffdiff2 from the cufflinks package [2] was used with the vOKA known annotation as reference. Cuffdiff2 yielded normalized expression levels for each gene as FPKM, and determined the differentially expressed genes between the quiescently and productively infected neurons (FDR<0.05). Visualization of the results was performed with IGV (Broad Institute).

[1] Langmead and Salzberg Nat Methods. 2012 9:357-9

[2] Trapnell et al Nat Biotechnol. 2013 31:46-53
